# Candidate Genes and Proteomic Biomarkers of Serum and Urine in Medication-Overuse Headache

**DOI:** 10.3390/ijms22169024

**Published:** 2021-08-21

**Authors:** Natalia A. Shnayder, Victoria B. Sharavii, Marina M. Petrova, Polina V. Moskaleva, Elena A. Pozhilenkova, Darya S. Kaskaeva, Olga. V. Tutynina, Tatiana E. Popova, Natalia P. Garganeeva, Regina F. Nasyrova

**Affiliations:** 1 The Center of Personalized Psychiatry and Neurology, V. M. Bekhterev National Medical Research Center for Neurology and Psychiatry, 192019 Saint-Petersburg, Russia; nreginaf77@gmail.com; 2 The Center of Collective Usage “Molecular and Cell Technologies”, V. F. Voino-Yasenetsky Krasnoyarsk State Medical University, 660022 Krasnoyarsk, Russia; stk99@yandex.ru (M.M.P.); polina-moscaleva@yandex.ru (P.V.M.); elena.a.pozhilenkova@gmail.com (E.A.P.); dashakas.ru@mail.ru (D.S.K.); tutynina_lelya@mail.ru (O.V.T.); 3 The International School Medicine of the Future, I. M. Sechenov First Moscow State Medical University (Sechenov University), 119991 Moscow, Russia; 4 The Yakutsk Scientific Center for Complex Medicine Problems, The Department of Epidemiology of Non-Infectious Diseases, 677018 Yakutsk, Russia; tata2504@yandex.ru; 5 The Department of General Medical Practice and Polyclinic Therapy, The Siberian State Medical University, 634050 Tomsk, Russia; garganeeva@gmail.com

**Keywords:** headache, chronic headache, proteomics, proteogenomics, serum biomarker, urine biomarker

## Abstract

Chronic headache is a topical problem of neurology, psychiatry and general practice. The medication-overuse headache (MOH) is one of the leading pathologies in the structure of chronic headache. However, early diagnosis of the MOH is challenging. We analyzed potential proteomic biomarkers of serum and urine in patients with MOH. Methods: We searched PubMed, Springer, Scopus, Web of Science, ClinicalKey, and Google Scholar databases for English publications over the past 10 years using keywords and their combinations. Results: We found and analyzed seven studies that met the search criteria for the purpose of the review, including 24 serum proteomic biomarkers and 25 urine proteomic biomarkers of MOH. Moreover, the candidate genes and locus of the studied serum (vitamin D-binding protein, lipocalin-type prostaglandin D2 synthase, apolipoprotein E, etc.) and urine proteomic biomarkers (uromodulin, alpha-1-microglobulin, zinc-alpha-2-glycoprotein, etc.) of MOH are presented in this review. Conclusions: The serum and urine proteomic biomarkers of MOH can potentially help with the identification of patients with MOH development. Due to the relevance of the problem, the authors believe that further investigation of the MOH proteomic biomarkers in different ethnic and racial groups of patients with primary headache is necessary. In addition, it is important to investigate whether medications of different drug classes influence the levels of serum and urine proteomic biomarkers.

## 1. Introduction

Chronic pain (CP) is an essential problem in healthcare. Approximately 20% of the European population is affected by CP [1], which has a significant influence on their daily social and working lives. Moreover, the economic impact of CP is more than heart disease, cancer and diabetes put together [2]. It is worth mentioning that among chronic painful disorders, headaches are predominant. According to world literature, with an incidence of 3% per year, 4–5% of the general population suffers from chronic headache, known as headache occurrence ≥15 days per month [3,4,5]. Chronic forms of headache, such as chronic migraine or chronic tension-type headache, often involve a high and daily intake of combination analgesics and acute headache medications (AHMs), such as nonsteroidal anti-inflammatory drugs (NSAIDs) and triptans [6]. Paradoxically, they only worsen the chronic symptoms, resulting in the development of the secondary headache, so-called medication-overuse headache (MOH) [7]. In the latest and current Third Edition of the International Classification of Headache Disorders (IHS ICHD-3), MOH, also known as a rebound headache, is described as a headache that is present on 15 or more days per month developing as a consequence of regular overuse of acute or symptomatic headache medication. Criterions of MOH: the overuse of simple analgesics on 15 or more days per month; or else the overuse of triptans, ergotamines, analgesics, opioids, and (caffeine or codeine-containing) combined analgesics on 10 or more days per month, for more than 3 consecutive months [8]. Although the prevalence of MOH in the general population is around 1–2% [9,10], MOH is still defined as a socio-economic burden worldwide, especially in lesser developed countries [11], associated with significant long-term morbidity, disability, and lower quality of life. According to systematic reviews of MOH epidemiology, it is most predominant in middle-aged women from 30 to 50 years old, with the male to female ratio around 1 to 3–4 [9,12,13,14,15]. The pathophysiological mechanisms of MOH development are still an ongoing debate. Nevertheless, frequent and regular consumption of acute headache medication does not seem enough to cause MOH, therefore individual predisposition and specifics of the medication class combined play a significant role in its development [16].

The proteomic analysis and the exploration of proteomic biomarkers are crucial in understanding a lot of medical conditions, especially cancer, cardiovascular and neurodegenerative diseases [17,18,19]. Thus, identification of chronic pain biomarkers can be helpful and valuable for clinicians in the diagnosis of patients at risk or with an already developed disorder, reducing the time and costs, selection of rational personalized pain treatment, and understanding of the underlying pathophysiological mechanisms of chronic pain development [20]. Moreover, a considerable number of patients with MOH can hide the truth from the doctor about the frequency and daily amount of acute headache medications they are consuming. Besides, the MOH phenotype is almost indistinguishable from other chronic headache phenotypes [11]. Therefore, it becomes even more challenging to diagnose MOH and selecting and monitoring the headache treatment. Furthermore, acute headache medication overuse leads to side effects, such as nephrotoxicity and kidney damage, gastrointestinal bleeding, liver impairment [11], especially in a group of patients who abuse NSAIDs [21]. It is not possible to prevent the development of renal impairment and acute renal failure caused by drug-induced nephrotoxicity by using traditional laboratory analyses, such as creatinine, creatinine clearance, urea, electrolytes, urine sediment [22]. However, urinary proteomics allows the potential risks of developing severe drug-induced kidney damage to be minimized by its detection in the early stages during a normal clinical presence, particularly in NSAIDs abusers, using a panel of protein biomarkers each informing on the integrated aspects of glomerular, tubular, and interstitial function [23].

The underlying mechanisms of chronic pain pathophysiology still remain poorly understood. Proteomics is one of the most promising areas that can significantly contribute to pain chronicity research, its better understanding and management.

The aim of the thematic review was the search of the potential proteomic biomarkers of serum and urine in patients with MOH.

## 2. Materials and Methods

We searched PubMed, Springer, Scopus, Web of Science, Clinicalkeys, and Google Scholar databases for English-language publications over the past 10 years using such keywords and their combinations as “primary headache”, “migraine”, “tension-type headache”, “cluster headache”, “medication-overuse headache”, “proteomics”, “proteomic biomarkers”, “serum”, “urine”, “laboratory diagnostic”, and “mass-spectrometry”.

We considered studies published from 2011 to 2021 and identified publications devoted to the search for proteomic biomarkers in medication-overuse headache. Based on search criteria, only 7 publications were included in this thematic review: serum proteomic biomarkers—two publications (2019, 2020); urine proteomic biomarkers—five publications (2012–2016). In addition, earlier publications of historical interest were included in the review, including studies on the biological role of potential serum and urinary protein biomarkers of pain in humans.

## 3. Results

### 3.1. Candidate Serum Proteomic Biomarkers of Patients with Medication-Overuse Headache

The role of proteomic biomarkers in chronic pain development is derived primarily from animal models. Thus, Bellei et al. (2017) demonstrated overexpressed serum proteins, particularly alpha-1-macroglobulin (A1MG), vitamin D-binding protein (VDBP), apolipoprotein A1 (APOA1), apolipoprotein E (APOE), prostaglandin-H2-D-isomerase (PTGDS), transthyretin (TTR), which can be involved in the expression or mediation of chronic pain, on a rat model after chronic constriction injury (CCI) of the sciatic nerve [24]. Additionally, Vacca et al. (2014, 2016) demonstrated the possibilities of using proteome analysis on animal models in pain-related protein identification [25,26].

The search for potential headache chronicity biomarkers is still ongoing. Few studies have shown the potential biomarkers of headache chronicity caused by medication overuse, such as SNVs of the angiotensin-converting enzyme, brain-derived neurotropic factor, catechol-O-methyltransferase genes, altered neurotransmitters metabolism, neurophysiological and neuroimaging changes [27]. However, blood plasma and urine are still the perfect sources for a long-term monitoring of disease and treatment changes due to the ease in obtaining them in multiple samples. Additionally, serum and urine proteomic biomarkers are more attractive as they are easily measurable.

Thus, Pellesi et al. (2019) showed the overexpression of lipocalin-type prostaglandin D2 synthase (L-PGDS) in the serum of MOH patients compared to healthy controls (219.2 ± 58.2 vs. 188.7 ± 39.2 ng/mL, *p* = 0.0038) and a not significant increase of apolipoprotein A1 (APOA1) (49.8 ± 23.7 vs. 46.9 ± 17.5 μg/mL, *p* = 0.546). In contrast, vitamin D-binding protein (VDBP) (289.5 ± 88.7 vs. 343.7 ± 9 5.3 μg/mL, *p* = 0.0058) and apolipoprotein E (APOE) (63.9 ± 13.3 vs. 72.5 ± 17.3 μg/mL, *p* = 0.0042) levels were deficient in MOH patients compared to healthy individuals. This finding of a higher level of L-PGDS in a serum shows that it may be the proteomic biomarker of headache chronicity associated with excessive intake of AHMs. It is interesting to note that according to the prevalent acute headache medication, patients who assumed mixtures had a higher serum level of L-PGDS compared to those who assumed NSAIDs or triptans (266.0 ± 44.6 vs. 214.9 ± 53.5 and 209.4 ± 57.5 μg/mL) [28].

In later research, Pellesi et al. (2020) further investigated the role of the serum proteome in the biological mechanisms of headache chronicity on the example of MOH. Authors used monodimensional gel electrophoresis (SDS-PAGE) and detected four protein bands that were further identified by LC-MS/MS-QO system and had a 1.54-fold to 2.82-fold higher level in MOH patients compared to the healthy controls. Research has discovered proteins increased in MOH patients: alpha-1 antitrypsin (A1AT), hemopexin (HEMO), immunoglobulin heavy constant alpha 1 (IGHA1), alpha-1-acid glycoprotein 1 (A1AG1), apolipoprotein A-4 (APOA4), haptoglobin (HPT), retinol binding protein (RETBP), transthyretin (TTHY). Moreover, some of them were at unusually high concentrations. Thus, HEMO has 2.85-fold and A1AG1 has 3.75-fold increased serum protein levels in MOH patients compared to healthy patients. However, only one protein, immunoglobulin kappa constant (IGKC), was decreased in MOH patients in comparison with healthy controls (<1-fold ). These results show the potential use of this novel serum proteomic biomarker for predicting headache progression and chronicity, particularly associated with medication overuse [29].

Candidate serum proteomic biomarkers of MOH are shown in Table 1.

### 3.2. Candidate Urine Proteomic Biomarkers of Patients with Medication-Overuse Headache

Urine proteomic analysis of NSAIDs, mixtures and triptans abusers [31,32] was carried out by Bellei et al. (2012) using one-dimensional gel electrophoresis (1D-SDS-PAGE) and further protein identification by Q-TOF LC/MS. Researchers have found seven proteins oversecreted by the kidneys in MOH patients, especially NSAIDs abusers, compared to healthy controls: uromodulin (UROM), alpha-1-microglobulin (AMBP), zinc-alpha-2-glycoprotein (ZAZG), inter-alpha-trypsin heavy chain H4 (ITIH4), Ig kappa chain C region (IGKC), non-secretory ribonuclease (RNAS2), and cystatin C (CYTC) (OR = 49, 95% CI 2.53–948.67 vs. controls; OR = 11.6, 95% CI 0.92–147.57 vs. triptans and mixtures groups). UROM is the most evident protein which is present in MOH patients’ urine in high concentrations compared to controls (triptans group OD = 14,800 ± 2764 vs. NSAIDs group OD = 12,500 ± 1652 vs. mixtures group OD = 11,100 ± 982 vs. control group OD = 2205 ± 291). The results of this study present the urinary proteomic profile of MOH patients and show their potential role in the development of headache chronicity and a possible relation with renal damage [21].

In a further study, Bellei et al. (2013) extended the results of the previous study [21], using two-dimensional gel electrophoresis (2-DE) followed by Q-TOF LC/MS. Research has shown 21 overexcreted proteins in MOH patients compared to control subjects with an expression difference of >1.5 fold. Proteins identified in the previous study [21]: UROM, ITIH4, AMBP, IGKC, RNAS2, CYTC. In addition, novel urine proteins were overexpressed: serum albumin (ALBU), alpha-1-antitrypsin (A1AT), actin, cytoplasmic 1 (ACTB), apolipoprotein H (APOH), serpin B3 (SPB3), annexin A1 (ANXA1), prostaglandin-H2-D-isomerase (PTGDS), perlecan (fragment) (PGBM), transthyretin (TTHY), proactivator polypeptide (SAP), nuclear transport factor 2 (NTF2), fatty acid-binding protein (FABP5), beta-2-microglobulin (B2MG), protein S100-A11 (S10AB), protein S100-A8 (S10A8). According to the results of this study, urine proteome has been demonstrated to participate in the drug-induced glomerular and tubular damage potentially caused by drug-toxicity. Therefore, urinary proteomic biomarkers can possibly be markers of medication overuse [23].

In later research the overexcretion of urinary proteins in patients with MOH compared to healthy patients was confirmed by Bellei et al. (2015) using Western blot analysis and Enzyme-linked Immunosorbent Assay (ELISA) in addition to SDS-PAGE, 2-DE. As in all previous studies, UROM has been described as the most intense protein band expressed in all MOH patients (lane 2 = triptans, lane 3 = NSAIDs, lane 4 = mixture abusers). Other visible proteins are AMBP, PTGDS, CYTC [30].

Candidate urine proteomic biomarkers of MOH are shown in Table 2.

**Figure 1:**
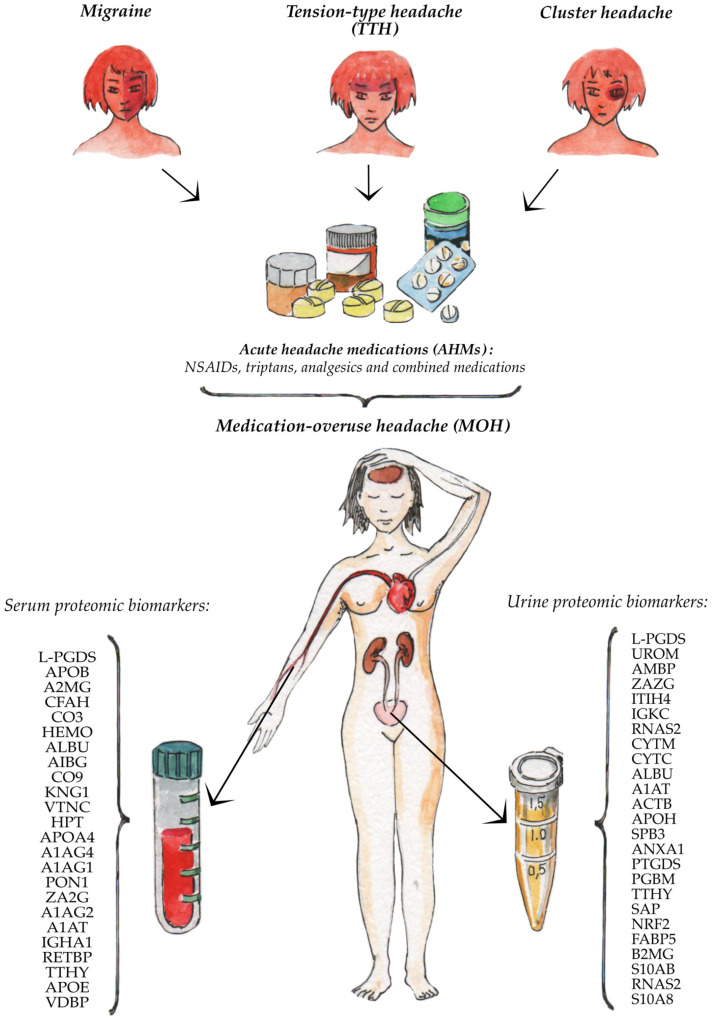
Serum and urine proteomic biomarkers of the medication-overuse headache.

The summary results of our thematic review are presented in Figure 1. 

We selected both serum and urinary proteomic MOH biomarkers. However, urinary MOH biomarkers may also be interesting from the standpoint of noninvasive sampling of biomaterial for analysis.

## 4. Discussion

MOH is a topical interdisciplinary medical, economic and social problem of modern healthcare [15,34]. A lot of psychological and socioeconomic factors are associated with MOH, such as low income and education [12,35,36,37,38], high prevalence of smoking, high body mass index [39] and sleeping problems [14], depression and anxiety [40,41], a family history of MOH or other substance abuse [42,43,44,45]. It is necessary to note that the availability of medications for patients with primary headaches (migraine, tension type headache, cluster headaches, etc.) also plays a huge role in the development of MOH. In some countries, NSAIDs, triptans, analgesics and combined analgesics containing opiates or barbiturates are available to patients in pharmacies without a prescription [46]. We believe that the regulation of prescriptions and usage of these medications should be mandatory. Diagnosis of primary headaches and MOH is based on the criteria of the current IHS ICHD-3 (2013) [8]. However, these criteria are mainly based on the patient’s medical history (anamnesis morbid and vitae) and detailed medication history, which are collected and clarified by talking to the patient. Unfortunately, patients who are abusing AHMs, such as NSAIDs, triptans, analgesics and combined medications may delay seeking for medical advice from a neurologist or general practitioner in a timely manner, because they ignore or underestimate the risk of MOH and its consequences, such as drug-induced depression, anxiety and sleep disorders [40,41], risk of suicide [47], behavioral disorders [48,49], physical and mental disability [40], reduced quality of life [50,51], and comorbid adverse drug reactions (ADRs): gastrointestinal bleeding [52], drug-induced nephrotoxicity [23,30,33], metabolic syndrome [53]. Therefore, it encourages researchers and clinicians to investigate novel and objective serum and urine biomarkers of MOH, including the proteomic biomarkers. Our review of the studies on human and animal models of MOH demonstrates an increasing interest in this problem, although up to this moment the number of studies is still small [21,23,24,28,29,30,33,54].

As diagnostic methods and laboratory diagnostic equipment are improving [55,56], the number of candidate proteomic serum and urine MOH biomarkers is increasing. On the one hand, it enhances the possibility of early diagnosis of MOH in the patients-at-risk group (e.g., patients with low compliance with the attending physician, self-medication, living in regions or countries with easy and free access to AHMs, such as NSAIDs, triptans, analgesics and combined medications in local pharmacies, etc.). On the other hand, the implementation of a large number of MOH proteomic biomarkers into real clinical practice may not be cost-effective. Conducting large multicenter studies among different ethnic groups is required in order to validate serum and urine MOH proteomic biomarkers and to develop personalized algorithms for their usage.

### 4.1. The Promising Serum and Urinary Proteomic Biomarkers of Medication-Overuse Headache

Based on our review, we would like to highlight only some of the serum and urine proteomic biomarkers that have been studied the most in recent years. Thus, lipocalin-type prostaglandin D2 synthase (L-PGDS) is both a serum and urine proteomic biomarker. Several studies showed that patients with MOH had it in high concentrations in both serum and urine [23,30]. L-PGDS is expressed in various tissues, such as the brain, retina, cochlea, and male reproductive organs, and it is found in different biological fluids, such as cerebrospinal fluid (CSF), ascites, seminal plasma, serum, urine, and amniotic fluid. Alongside hematopoietic-type PGDS (H-PGDS), L-PGDS is related to a group of prostaglandin D synthases (PGDS) responsible for converting PGH_2_ to PGD_2_ [57], which is involved in a variety of central nervous system (CNS) functions, such as sedation, nonrapid eye movement (NREM) sleep and PGD_2_-allodynia. In addition to synthesizing PGD_2_, a potent endogenous nociceptive modulator [58] within the cells, in the extracellular space and body fluids L-PGDS binds various small nonsubstrate lipophilic molecules such as retinal, retinoic acid [59], bilirubin, biliverdin [60], gangliosides [61], amyloid β peptides [62]. Therefore, L-PGDS can potentially be a promising proteomic biomarker as the entire prostaglandin system plays a huge role in pain and central sensitization development.

Vitamin D-binding protein (VDBP) is a monomeric glycoprotein synthesized and secreted predominantly by the liver. VDBP can be found in various body fluids, such as plasma, ascitic fluid, CSF. It was originally known as a group-specific component (GC) because of its worldwide polymorphisms [63]. It is also known as macrophage-activating factor (GcMAF/DBP-MAF) because it initiates macrophage activity, a key part of the host defense system [64]. VDBP has multiple functions, such as actin binding and neutrophil chemotaxis [65]. However, the main function of VDBP is to bind vitamin D and its plasma metabolites and transport them to target tissues. There are several studies showing the relation between Vitamin D deficiency or insufficiency and chronic headache development [66,67]. Possible mechanisms include the anti-inflammatory role of vitamin D, specifically, decreased production of inflammatory substances which activate trigeminal nerve, the main structure involved in migraine development [68,69]. Moreover, by inhibiting nitric oxide (NO) synthase expression, vitamin D reduces the production of NO, a key endogenous mediator in headaches, such as migraine [70] and tension-type headaches [71], deficient levels of VDBP in MOH patients can be associated with a decreased level of vitamin D, which can be one of the mechanisms of chronic headache development.

Apolipoprotein E (APOE) is a multifunctional protein which participates in lipid metabolism and carries lipids in different tissues of the body, including both the peripheral and the central nervous system [72]. APOE mediates the binding of APOE-containing lipoproteins and lipid complexes to specific cell-surface receptors, such as the low-density lipoprotein (LDL) receptors, the LDL receptor-related protein (LRP), the very low-density lipoproteins (VLDL) receptor, the APOE receptor-2, gp330. Moreover, *APOE* demonstrates genetic polymorphisms containing three common alleles, ε2, ε3, ε4 that encode three isoforms (APOE2, APOE3, APOE4) [73]. According to the available literature, NO synthesis is dependent on *APOE* polymorphisms. Thus, the *APOE4* gene is involved in the production of NO through increased uptake of arginine in the microglia compared to *APOE3* gene [74]. *APOE* polymorphisms also influence the expression of cytokines which play a huge role in inflammation, pain modulation and central sensitization [75].

Alpha-1 antitrypsin (A1AT), also known as alpha-1 proteinase inhibitor, is an acute phase reactant and serine protease inhibitor (serpin) whose targets are elastase, plasmin, thrombin, trypsin, chymotrypsin, plasminogen activator, and is mostly produced in the liver and expressed by hepatocytes [76]. It is important to note that A1AT inhibits NO production [76], a key molecule in the pathophysiology of primary headaches [77].

Hemopexin (HEMO) is an acute-phase plasma glycoprotein with the highest affinity to heme among all known proteins and is responsible for transporting heme from the plasma to the liver for breakdown and iron recovery. Moreover, it has intracellular antioxidant activities and, therefore, is involved in protecting cells from scavenging and oxidative stress. HEMO is expressed in various tissues such as the nervous system, skeletal muscle, retina, kidney, but mainly in the liver [78]. HEMO was found at significantly different levels in the CSF of patients with leptomeningeal metastases of breast cancer with neurological complications [79] and with other neuropathologically confirmed diseases [80].

Haptoglobin (HPT) is an acute-phase protein whose main function is to bind hemoglobin (Hb) during hemolysis, forming the Hb-Hp complex, which is crucial for the elimination of free Hb by the macrophage CD163 scavenger receptor expressed on the liver Kupfer cells surface, therefore preventing kidney damage. HPT also acts as an antioxidant and plays a huge role in the neutralization of oxidative stress.

Retinol binding protein (RETBP) is specific carrier protein whose only known function is to transport retinol (vitamin A) from hepatic stores to target tissues [81].

Transthyretin (TTHY) is a homotetrameric protein mostly produced in the liver and choroid plexus of the brain. The main function of TTHY, the transport of thyroxine and RETBP, is well-known. However, other functions of this protein, namely in the nervous system, have emerged.

Urinary biomarkers are increasingly used in the diagnosis, classification and prognosis of kidney diseases. Uromodulin (UROM), also known as Tamm-Horsfall protein, is the most abundant protein in urine. It can also be found in serum in lower amounts. UROM is exclusively produced by renal epithelial cells in the kidney. Amongst all the functions of UROM, the most important ones are the regulation of ion transport in the thick ascending limb, immunomodulation and protection against urinary tract infections and kidney stones [82]. In clinical practice, UROM is used as a valuable biomarker of tubular damage and kidney diseases, including drug-induced nephrotoxicity caused by medication overuse.

Alpha-1-microglobulin (AMBP) is one of the urinary microproteins that are becoming more and more important in clinical diagnostics and practice. AMBP is a tubular glycoprotein, mostly expressed in liver, blood and kidney, which is used for detecting acute lesions of proximal tubules. It was first discovered 40 years ago in human urine [83]. Functions of AMBP are still unknown. However, some reports have suggested that AMBP may be involved in oxidant-scavenging and have enzymatic reductase properties as an antioxidant [84]. Altered plasma and urine levels of AMBP are usually markers of impaired liver or kidney functions. Therefore, nowadays, urinary AMBP is considered as a promising inexpensive alternative biomarker for the early detection and diagnosis of urinary tract disorders [85], including kidney damage, caused by acute headache medication overuse.

Zinc-alpha-2-glycoprotein (ZAZG) is a single-chain polypeptide secreted in various body fluids, such as serum and urine. Despite the fact that functions of ZAZG still remain unknown, some reports suggest that ZAZG has a lot of important functions in the human body, including fertilization and lipid mobilization, therefore it is considered as a multidisciplinary protein [86,87]. As its structural organization and folding characteristics are similar to the MHC class I antigen-presenting molecule, it may have a biological role in the immune response. ZAZG is used as a tumor biomarker for various carcinomas [87]. However, some immunohistochemical analyses have shown predominant expression in the kidney tubules of the human [88]. Therefore, urinary ZAZG may be a potential biomarker of renal damage, including drug-induced nephrotoxicity.

Inter-alpha-trypsin heavy chain H4 (ITIH4) is a liver-produced glycoprotein belonging to the liver-restricted serine protease inhibitor family. Its biological role is still unknown, as ITIH4 is cleaved in a number of different pathologies. However, it plays important role in various biological processes, such as inflammatory responses to trauma, liver formation or regeneration [89].

Immunoglobulin kappa constant (IGKC) is a constant region of immunoglobulin light chains, also known as antibodies, membrane-bound or secreted glycoproteins produced by B lymphocytes. The main function of IGKC is to serve as receptors which, upon the binding of a specific antigen, trigger the clonal expansion and differentiation of B lymphocytes into immunoglobulin-secreting plasma cells. Secreted immunoglobulins play a significant role in the mediation of the effector phase of humoral immunity, which results in the elimination of bound antigens [90].

Nonsecretory ribonuclease (RNAS2) is a pyrimidine specific nuclease with a slight preference for cytotoxin and helminthotoxin. RNAS2 is selectively chemotactic for dendritic cells and possesses a wide variety of biological activities.

Cystatin C (CYTC) is a nonglycosylated basic protein encoded by the CST3 gene found in all nucleated cells. CYTC was first discovered in 1961 and formally named in 1984. CYTC is a potent inhibitor of lysosomal proteinases and extracellular inhibitors of cysteine proteases that play a huge role in human vascular pathophysiology [91]. Nowadays, CYCT is used as a more accurate alternative to serum creatinine for measuring glomerular filtration rate (GFR), one of the main parameters in the estimation of kidney function [92]. CYCT is increasingly used as an earlier biomarker for acute kidney injury, a superior marker of kidney transplant function, cardiovascular disease risk and transplant failure. Therefore, CYTC may be a promising proteomic biomarker for MOH and drug-induced nephrotoxicity, caused by acute headache medication overuse.

The serum and urine proteomic biomarkers of patients with MOH that we discussed in this review are promising for clinical applications in neurological and psychiatric practice. Their number is increasing. Therefore, it requires further investigation and validation of these proteomic biomarkers by conducting large multicenter clinical studies in different ethnic groups. Apparently, various medications differently influence the synthesis and excretion of these proteomic biomarkers, depending on their drug class. However, this problem currently has not been studied sufficiently yet and is still controversial. Nevertheless, the implementation of the results of fundamental studies into the clinical practice in clinical pharmacology, neurology and psychiatry, will significantly improve the differential diagnosis of primary headaches and MOH, revise current clinical criteria for MOH diagnosis, and, most importantly, modify the management of patients with primary headaches.

So, proteomics is a powerful tool to identify serum and urinary proteins, potential biomarkers, which can contribute to a better understanding of the mechanisms of the diseases. Moreover, the urine protein composition and fragmentation are more stable than in plasma or serum, which are vulnerable to proteolysis during and after sampling [32].

### 4.2. Limitations

There are several limitations in our thematic research. We studied only English-language publications. It is likely that taking different medications can have a variable effect on the increase in the level of various proteomic biomarkers in serum and/or urine. Men and women may respond differently to the development of a headache and have different levels of studied proteomic biomarkers of MOH. Further studies are needed to study the effect of modifiable (medications for primary headache treatment in monotherapy and polytherapy, comorbid diseases, dietary habits, etc.) and unmodifiable (gender, age, genetic predisposition) predictors on the level of previously studied proteomic MOH biomarkers in humans. Further studies are needed to investigate the effects of carriage of ONVs/polymorphisms of genes encoding serum and urinary proteomic biomarkers on serum and urine levels in patients with MOH. Further research is needed to confirm the role of mutations of genes encoding proteomic MOH biomarkers using knockout mice. Finally, the specific serum and urinary proteomic MH biomarkers analyzed by us are difficult to implement in real clinical practice due to their large number and high cost of laboratory diagnostics.

### 4.3. Summary

Primary headache disorders are one of the topical problems in modern neurological, psychiatric and general practice. Most likely this is due to the high prevalence of primary headache in young working age people [46,93] with a high occurrence of self-treatment. Early diagnosis and differential diagnosis of MOH is challenging because patients may use drugs without a doctor’s prescription (self-treatment) for a long period of time and not seek medical care from physicians. Moreover, they can conceal abuse of NSAIDs, triptans, analgesics and combined medications (ergotamines, opioids, barbiturates, benzodiazepines, etc.). MOH is probably one of the most costly headache disorders for both society and the sufferer [15,94]. For example, the national costs for MOH were estimated EUR 5–10 billion in Italy, Spain and France [94]. The 3-month cost per patient with MOH fell from EUR 2989 to EUR 1160 in Italy [95]. Moreover, the total cost for MOH patients is USD 10,179,000,000 annually in Iran [96]. A high economic burden for MOH was shown in other countries in the world as well [46,97,98,99]. Thus, the worldwide personal and economic costs are enormous [15,100].

We identified and analyzed seven studies that met the search criteria for the purposes of the thematic review, including 24 serum proteomic biomarkers and 25 urinary proteomic headache biomarkers. In addition, the review presented candidate genes for the studied serum (VDBP, LTPD2S, APOE, etc.) and urinary (UROM, AMBP, ZAZG, etc.) proteomic biomarkers. Overall, however, the results indicate that further research is needed to confirm the beneficial effects of these serum and urinary biomarkers on the early diagnostics of MOH in adults. In addition, it is important to investigate whether medications of a different drug class influence the level of serum and urine proteomic biomarkers.

## 5. Conclusions

This thematic review showed that proteomic biomarkers in serum and urine could potentially aid in the identification of patients with MOH. However, research into this problem is at the beginning of its journey. Further research is needed in the future to be able to translate the results into clinical practice.

## Figures and Tables

**Table 1:** Candidate serum proteomic biomarkers of medication-overuse headache.

Protein Full Name	Entry Name	Gene Name	Locus	Protein Main Function	Theor. Mass.	References
Lipocalin-type prostaglandin D2 synthase	L-PGDS	*PTGDS*	9q34.3	Prostaglandin biosynthesis process	21,029	[23,30]
Apolipoprotein B100	APOB	*APOB*	2p24.1	Cholesterol metabolism	516,651	[23,30]
Alpha-2-macroglobulin	A2MG	*A2M*	12p13.31	Enzyme binding	164,613	[23,30]
Complement factor H	CFAH	*CFH*	1q31.3	Complement activation	143,480	[23,30]
Complement C3 (fragm)	CO3	*C3*	19p13.3	Complement activation	188,569	[23,30]
Hemopexin	HEMO	*HPX*	11p15.4	Metal ion binding	52,385	[23,30]
Serum albumin	ALBU	*ALB*	4q13.3	Metal binding	71,317	[23,30]
Alpha-1B-glycoprotein	AIBG	*A1BG*	19q13.43	Neutrophil, platelet degranulation	54,790	[23,30]
Complement component C9	CO9	*C9*	5p13.1	Complement activation	64,615	[23,30]
Kininogen-1	KNG1	*KNG1*	3q27.3	Cysteine-type endopeptidase inhibitor activity	72,996	[23,30]
Vitronectin	VTNC	*VTN*	17q11.2	Heparin binding	55,069	[23,30]
Haptoglobin	HPT	*HP*	16q22.2	Acute phase response	45,861	[23,30]
Apolipoprotein A-4	APOA4	*APOA4*	11q23.3	Lipid binding	45,371	[23,30]
Alpha-1-acid glycoprotein 1	A1AG1	*ORM1*	9q32	Inflammatory response	23,725	[23,30]
Serum paraoxonase/arylesterase 1	PON1	*PON1*	7q21.3	Hydrolase	39,877	[23,30]
Zinc-alpha-2-glycoprotein	ZA2G	*AZGP1*	7q22.1	Protein transmembrane transporter activity	34,465	[23,30]
Alpha-1-acid glycoprotein 2	A1AG2	*ORM2*	9q32	Acute phase response	23,873	[23,30]
Alpha-1-antitrypsin	A1AT	*SERPINA1*	14q32.13	Protease inhibitor	46,737	[23,30]
Immunoglobulin heavy constant alpha 1	IGHA1	*IGHA1*	14q32.33	Antigen binding	37,655	[23,30]
Retinol-binding protein	RETBP	*RBP4*	10q23.33	Retinol binding	23,010	[23,30]
Transthyretin	TTHY	*TTR*	18q12.1	Hormone binding	15,887	[23,30]
Apolipoprotein E	APOE	*APOE*	19q13.32	Lipid transport	36,154	[23,30]
Vitamin D-binding protein	VDBP	*GC*	4q13.3	Vitamin D transport	52,918	[23,30]

Protein entry name, according to the UniProtKB database. Theoretical molecular weight (Da).

**Table 2:** Candidate urine proteomic biomarkers of medication-overuse headache.

Protein Full Name	Entry Name	Gene Name	Locus	Protein Main Function	Theor. Mass.	References
Lipocalin-type prostaglandin D2 synthase	L-PGDS	*PTGDS*	9q34.3	Prostaglandin biosynthesis process	21,029	[23,30]
Uromodulin (or Tamm-Horsfall urinary glycoprotein)	UROM	*UMOD*	16p12.3	Cellular defense response	69,761	[21,23,30,33]
Alpha-1-microglobulin	AMBP	*AMBP*	9q32	Calcium channel inhibitor activity	38,999	[21,23,33]
Zinc-alpha-2-glycoprotein	ZAZG	*AZGP1*	7q22.1	Protein binding	34,259	[21]
Inter-alpha-trypsin heavy chain H4	ITIH4	*ITIH4*	3p21.1	Acute-phase response	103,357	[21,23,33]
Ig kappa chain C region	IGKC	*IGKC*	2p11.2	Complement activation	11,765	[21,23,33]
Non-secretory ribonuclease	RNAS2	*RNASE2*	14q11.2	Chemotaxis	18,354	[21]
Cystatin M	CYTM	*CST6*	11q13.1	Cystein-type endopeptidase inhibitor activity	16,511	[21]
Cystatin C	CYTC	*CST3*	20p11.21	Cystein-type endopeptidase inhibitor activity	15,799	[21,30,33]
Serum albumin	ALBU	*ALB*	4q13.3	Metal binding	69,367	[23,33]
Alpha-1-antitrypsin	A1AT	*SERPINA1*	14q32.13	Protease inhibitor	46,737	[23,33]
Actin, cytoplasmic 1	ACTB	*ACTB*	7p22.1	Cell junction assembly	41,737	[23,33]
Apolipoprotein H	APOH	*APOH*	17q24.2	Heparin binding	38,298	[23,33]
Serpin B3	SPB3	*SERPINB3*	18q21.33	Cystein-type endopeptidase inhibitor activity	44,565	[23,33]
Annexin A1	ANXA1	*ANXA1*	9q21.13	Calcium ion binding	38,714	[23,33]
Prostaglandin-H2-D-isomerase	PTGDS	*PTGDS*	9q34.3	Prostaglandin biosynthesis process	21,029	[23,30,33]
Perlecan (fragment)	PGBM	*HSPG2*	1p36.12	Angiogenesis	468,830	[23,33]
Transthyretin	TTHY	*TTR*	18q12.1	Protein binding	15,887	[23,33]
Proactivator polypeptide	SAP	*PSAP*	10q22.1	Enzyme activator activity	58,113	[23,33]
Nuclear transport factor 2	NTF2	*NUTF2*	16q22.1	Positive regulation of protein import into nucleus	14,478	[23,33]
Fatty acid-binding protein	FABP5	*FABP5*	8q21.13	Fatty acid binding	15,164	[23,33]
Beta-2-microglobulin	B2MG	*B2M*	15q21.1	Antigen processing and presentation of endogenous peptide antigen via MHC class I	13,715	[23,33]
Protein S100-A11	S10AB	*S100A11*	1q21.3	Calcium ion binding	11,740	[23,33]
Non-secretory ribonuclease	RNAS2	*RNASE2*	14q11.2	Chemotaxis	18,354	[23,33]
Protein S100-A8	S10A8	*S100A8*	1q21.3	Calcium ion binding	10,835	[23,33]

Protein entry name, according to the UniProtKB database. Theoretical molecular weight (Da).
